# Supplementary material for: Barriers and facilitators of adherence to social distancing recommendations during COVID-19 among a large international sample of adults
Source: PLoS One. 2020 Oct 7;15(10):e0239795. doi: 10.1371/journal.pone.0239795 (PMC7540845; doi:10.1371/journal.pone.0239795)
Supplement: Attachment — Submitted filename: PONE-D-20-13845_r2_comments.pdf [file pone.0239795.s002.pdf]

Comments – PONE-D-20-13845

This is my second review of this manuscript. Below I comment on the authors' responses to each point I made in my first review and include some new comments. Original comments are in black text, and comments part of this review are in blue text.

Major points

Background

The paper could benefit from having a diagram of the conceptual model (i.e. last paragraph of the background section) that can help frame the paper.

Satisfactory.

The authors say "Finally, given that men are more likely to die from COVID and older adults are at higher risk of being infected by Sars-Cov-2, it is likely that gender and age could differentially impact adherence to social distancing behaviours.". Can the author cite studies showing that men are more likely to die from COVID-19 and older adults are at higher risk of being infected by Sars-Cov-2? Also, I wonder if the fact that men die more than women and that older adults are more likely to be infected compared to their younger counterparts is the only reason why gender and age could impact adherence to social distancing behaviours differently. It would be good if the authors could expand on the other plausible reasons why we could find heterogeneous results. What does the literature tell about women behaving more cautiously than men, and why may adherence to preventative health behaviours vary by sex and age? Also, if men are more likely to die from COVID-19, they are likely to be more adherent to social distancing recommendations, but even the authors find that men are less adherent to social distancing recommendations.

Partially addressed. The authors have not commented on the last issue 'If men are more likely to die from COVID-19, they are likely to be more adherent to social distancing recommendations, but even the authors find that men are less adherent to social distancing recommendations', and I think they could provide some explanations for why they find that men are less adherent to social distancing recommendations.

The authors say "In the context of the COVID-19 pandemic, it seems reasonable to assume that individual reasons to adhere to social distancing measures (e.g., desire to protect self and others) as well as external circumstances or motivators (e.g., workplace/school conducted remotely) contribute to engagement in and adherence to preventative behaviours, such as social distancing. In addition, individual characteristics, such as demographic and psychological profile (educational level, health literacy, anxiety/stress, empathy towards others) might also play a role in adherence. Finally, given that men are

more likely to die from COVID and older adults are at higher risk of being infected by Sars-Cov-2, it is likely that gender and age could differentially impact adherence to social distancing behaviours.”. One factor that is lacking in this paragraph is the family. An individual is part of a family. An individual may live with their partner, their kids, etc. They may also live with the most vulnerable people in this pandemic, such as their old parents or a partner with a pre-existing illness. The family composition may be an important socio-demographic predictor of social distancing behavioural outcomes. It would be good to include this factor in the analysis, or, if not available, at least discuss it in the background section.

Partially addressed. A discussion of this factor is still lacking in the background section. The authors should consider adding this factor to the paragraph discussing the sociodemographic variables that might play a role in adherence to social and physical distancing.

Methods

The survey was piloted on 15 individuals whose data were not included in the analysis. It would be good to mention what if this is in line with what is usually done in the literature. Are surveys usually piloted on more/less than 15 individuals? And, what was their assessment of the survey, did they find it easy to complete?

Satisfactory.

I could not find the list of motivations for social distancing and social distancing behaviours in this section. The authors should here refer to Table 2 and Table 3 from the results section to allow the reader to know the motivations for social distancing and social distancing behaviours.

Satisfactory.

P. 9 The authors conceptualised adherence to social distancing as “always” endorsing the behaviour (coded as “1”) and nonadherence as behaviour endorsed less often than “always”, including “never”, “sometimes”, or “often” response choices (coded as “0”). It would be good to specify why “never” was treated the same way as “sometimes” and “often” and what this could imply for your results. If the reason is purely methodological, then I wonder why not using a tobit model. If conceptual, please specify. Also, please give an example of a behaviour where the “not applicable” option could be used.

Partially addressed. The authors have not commented on the last issue: “please give an example of a behaviour where the “not applicable” option could be used.”

Does the model include variables for country of residence? Because countries took different approaches (even within the same category ‘moderate rules’ / ‘strict rules’, there are differences in the measures adopted), the behaviours may also vary by country.

Partially addressed. Measures taken were different across countries even when countries fell under the same category, and this should be taken into account in the model, are results robust to including country fixed effects (as opposed to countries with strictly enforced guidelines for social distancing etc.)?

P. 9 The authors say: “During data collection, recommendations and policies for social distancing differed by region or country but did not change within one region or country, hence our regression models did not account for timing of survey completion.”. If, on the one hand, recommendations and policies did not change, on the other, the number of cases and deaths have increased over the period of analysis and this might have changed people’s behaviours by for example increasing their adherence to the social distancing measures. It would be good if the authors could account for the passage of time in their analysis.

Not addressed. The passage of time should be taken into account in the model estimation, or the authors should at least check that results are robust to this inclusion.

Results

The results section is very difficult to follow because the results are presented as if they were reported on a presentation with bullet points. The whole section is organised in a similar fashion: “Endorsement rates for the four sets of motivations “for” (facilitators) and “against” (barriers) social distancing are included in Table 2. Highest endorsement rates were found for the following facilitators of social distancing: “I want to protect myself” (84%) and “I want to avoid spreading the virus to others” (83%) (individual-level facilitators); “I want to protect others” (86%) and “I feel a sense of responsibility to protect our community” (84%) (interpersonal-level); “My workplace/ school recommended we practice social distancing” (54%) and “My workplace /school conducts operations remotely” (51%) (organizational-level); “Restaurants in my area are closed for eating-in” (95%) and “Community centers and recreational facilities in my area are closed” (94%) (community-level).”. The authors should find a better way to present the results because the way it stands now is not ok.

Satisfactory.

The organizational-level motivations against social distancing stand out for having the lowest endorsement rates, i.e. “My workplace/ school recommended we practice social distancing” (54%) and “My workplace /school conducts operations remotely” (51%). Can the authors speculate why we get such low rates in this cluster?

Satisfactory.

Limitations

The implications of the limitations should be discussed in the paper. The authors mention three limitations of their study, but do not discuss their implications. Among them, the issue related to the sample selection is the most important. The authors cannot do much about it, and I think that they have been clear about the fact that the sample is not representative of the general population. On the other hand, I think the authors should at least discuss what are the implications of using this sample. If possible and sensible, I recommend having a table that compares the sample characteristics to the characteristics of the general population; this way we could at least know how the sample differs from the population.

I agree with the authors that they had already discussed their limitations, as I also acknowledged in my previous comment. My point was about the implications of the limitations that could be further discussed. For example, what does the fact you are missing part of the population mean in terms of your results, e.g. how do you think the endorsement rates might change?

Minor points

The authors say one of the social distancing measures is "maintaining a 2-metre distance between self and others when in public". It would be good to specify what 'when in public' means. In particular, does social distancing apply to the private sphere too? For example, if I am visiting my parents at their home, do we still have to maintain the distance? Also, social distancing varies across countries from two metres down to one metre. It would be good to either be more general and say "at least a 1-metre distance", or if the 2-metre rule is kept be more specific about where.

Satisfactory.

P.5 The authors say: "Since the World Health Organization (WHO) declared COVID-19 a pandemic on March 11, 2020, national and international public health agencies proposed several measures to contain or mitigate the virus transmission ranging from complete quarantine of the population of an entire region, as in Wuhan, China (virus containment) to various degrees of social distancing measures coupled with rigorous personal hygiene (e.g., washing hands frequently and thoroughly, avoiding touching the eyes, nose, and mouth, coughing and sneezing into the elbow; wearing face masks when in public) in Canada, the United States, and Europe (mitigation of transmission).". The response was not the same across Europe, in fact some European governments imposed a national quarantine. On 9 March, i.e. two days before WHO announced COVID-19 outbreak a

pandemic, the Italian government imposed a national quarantine like the Chinese government did in Wuhan. Italy was not the only one, others followed, e.g. Greece.

Not addressed. I would suggest the authors say "…in Canada, the United States, and some European countries (mitigation of transmission)."

P.6 "Finally, given that men are more likely to die from COVID" should be "Finally, given that men are more likely to die from COVID-19".

Satisfactory.
