# Supplementary material for: Barriers and facilitators of adherence to social distancing recommendations during COVID-19 among a large international sample of adults
Source: PLoS One. 2020 Oct 7;15(10):e0239795. doi: 10.1371/journal.pone.0239795 (PMC7540845; doi:10.1371/journal.pone.0239795)
Supplement: Attachment — Submitted filename: Reviewers Comments.2020.08.010.docx [file pone.0239795.s001.docx]

**Response to Reviewers’ Comments: PONE-D-20-13845**

Dear Dr. Capraro

Thank you for the positive outlook on our manuscript and for your thoughtful comments and suggestions. We are grateful for the opportunity to revise our work. We have made substantial revisions to the manuscript based on these suggestions. Please see below for detailed responses to Editor and Reviewer’s comments, in addition to a summary of changes made to the manuscript.

**EDITOR COMMENTS**

**Editor’s Comment #1**: I have now collected one review from one expert in the field. I was unable to find a second reviewer. However, the review I could collect is very detailed, and I am myself familiar with the emerging literature on Covid-19, therefore I feel comfortable in making a decision with only one review. The review is positive and suggests major revision. I agree with the reviewer and therefore I would like to invite you to revise your work for Plos One.

Apart from the reviewer's comments, I would like to suggest another improvement. While reading the manuscript, I had the feeling that the emerging literature on Covid-19 was largely neglected. This point should be improved. A good starting point is Van Bavel et al.'s "perspective article" on what social and behavioral science can do to promote pandemic response, published in Nature Human Behaviour.

**Our response to Editor’s comment #1:** We thank the editor for pointing out this limitation, and greatly appreciate their suggestions for relevant literature. We added reference to the Van Bavel et al, 2020 review as well as another review of behavioural science strategies, which can be used for COVID-19 prevention interventions, Bonell et al, 2020.

**Editor’s Comment #2**: Also, there have been several works testing which appeals and messages promote pandemic response (Bilancini et al. 2020; Capraro & Barcelo, 2020a; Capraro & Barcelo, 2020b; Everett et al. 2020; Heffner et al. 2020; Jordan et al. 2020). Of course it is not a requirement to cite exactly these works, but in any case I think that you should do a much better job at placing your work within the current academic discussion.
Looking forward for the revision.

**Our response to Editor’s Comment #2:** We have reviewed each suggestion carefully and searched the literature for newer articles and pre-prints. We have restructured the introduction substantially and incorporated the works that we believed were related to our study rationale and aims (Heffner et al. 2020; Jordan et al. 2020 and others). We hope the editor is satisfied with this revised version.

**REVIEWER #1: COMMENTS – PONE-D-20-13845 - LILIANA ANDRIANO**

This paper describes the most frequently endorsed motivations to engage in social distancing and the rates of adherence to social distancing recommendations and examines the predictors of adherence to social distancing recommendations, using cross-sectional data collected online on more than 2,000 English-speaking adults from Europe and North America. The topic is undoubtedly relevant, and the findings are very important. However, the statistical approach can be improved, and the results section is difficult to read making the results hard to digest at first. I have a number of questions and suggestions for edits/adjustments. Below are some major and minor points.

We would like to thank Dr. Andriano for the thoughtful comments on our manuscript and we hope that we addressed the points raised in a satisfactory manner.

**Major points**

**Reviewer #1, Comment #1:** “Background - The paper could benefit from having a diagram of the conceptual model (i.e. last paragraph of the background section) that can help frame the paper.

**Our response to Reviewer #1, Comment #1:** We included a graphic representation of the social-ecological model and a list of the motivation items, as circumscribed to each level of the model, in Appendix 1. We added the following text to the Method section, Measures subsection, Motivations for Social Distancing paragraph:

- “*A complete list of motivation items, as circumscribed to the levels of the social-ecological model, is included in Table 2 of the results. A graphical representation of the social-ecological model was included in Appendix 1.”*

We did not think it was necessary to include graphical representations of the various models that use motivation to predict/explain health behaviour change- described briefly in the introduction- since those models were mentioned primarily to provide a rationale for why ‘motivation’ is a key construct to study relative to adherence to health behaviors.

**Reviewer #1, Comment #2:** “Background - The authors say “Finally, given that men are more likely to die from COVID and older adults are at higher risk of being infected by Sars-Cov-2, it is likely that gender and age could differentially impact adherence to social distancing behaviours.” Can the author cite studies showing that men are more likely to die from COVID-19 and older adults are at higher risk of being infected by Sars-Cov-2?

**Our response to Reviewer #1, Comment #2:** We included references for the socio-demographic variables that are associated with higher mortality from COVID-19:

- *“In addition, sociodemographic variables, such as gender, age, socioeconomic and minority status, as well as health status, might also play a role in adherence to social and physical distancing, as it has been found that mortality from and severity of COVID-19 is higher among men (23,24), older individuals (24,25), those with predisposing conditions (25), and racial minorities. For example, in a Southern state of the United States with a population of 31% Black, hospitalization rate was 77% and mortality rate was 71% for Blacks compared to whites (26).”*

**Reviewer #1, Comment #3:** Background - Also, I wonder if the fact that men die more than women and that older adults are more likely to be infected compared to their younger counterparts is the only reason why gender and age could impact adherence to social distancing behaviours differently. It would be good if the authors could expand on the other plausible reasons why we could find heterogeneous results. What does the literature tell about women behaving more cautiously than men, and why may adherence to preventative health behaviours vary by sex and age? Also, if men are more likely to die from COVID-19, they are likely to be more adherent to social distancing recommendations, but even the authors find that men are less adherent to social distancing recommendations.

**Our response to Reviewer #1, Comment #3:** We reported in the Introduction on some of the socio-demographic variables that have been associated with higher mortality from COVID-19 (see response to comment #2 above), but did not intend to investigate the mechanisms driving those associations. Further, this is an exploratory and descriptive study and as such, we did not have (directional) hypotheses, so we found it unnecessary at this point in time to speculate on how certain demographic variables might relate to social distancing behaviors.

**Reviewer #1, Comment #4:** Background -The authors say “In the context of the COVID-19 pandemic, it seems reasonable to assume that individual reasons to adhere to social distancing measures (e.g., desire to protect self and others) as well as external circumstances or motivators (e.g., workplace/school conducted remotely) contribute to engagement in and adherence to preventative behaviours, such as social distancing. In addition, individual characteristics, such as demographic and psychological profile (educational level, health literacy, anxiety/stress, empathy towards others) might also play a role in adherence. Finally, given that men are more likely to die from COVID and older adults are at higher risk of being infected by Sars-Cov-2, it is likely that gender and age could differentially impact adherence to social distancing behaviours.”.

One factor that is lacking in this paragraph is the family. An individual is part of a family. An individual may live with their partner, their kids, etc. They may also live with the most vulnerable people in this pandemic, such as their old parents or a partner with a pre-existing illness. The family composition may be an important socio-demographic predictor of social distancing behavioural outcomes. It would be good to include this factor in the analysis, or, if not available, at least discuss it in the background section.

**Our response to Reviewer #1, Comment #4:** The reviewer is correct in stating that living arrangements/family composition is an important variable to address. While we did not assess this as part of the demographic survey, we had conceptualized “having or living with a vulnerable family member” as interpersonal motivations, reflected in several items:

- *Items #24: “I have friends or family who are vulnerable to the virus.”*
- *item # 26: “I live with someone who is vulnerable to the virus.”*
- *Item #29: “I don’t have friends or family who are vulnerable to the virus.”*

**Reviewer #1, Comment #5:** Methods - The survey was piloted on 15 individuals whose data were not included in the analysis. It would be good to mention what if this is in line with what is usually done in the literature. Are surveys usually piloted on more/less than 15 individuals? And, what was their assessment of the survey, did they find it easy to complete?

**Our response to Reviewer #1, Comment #5:** We added the following text to the Method section, Participants and Procedures subsection:

- *“A preliminary version of the online survey was piloted on 15 individuals whose data were not included in this report and who provided edits to the items to improve readability, suggestions for the question flow, and corrections for small grammatical errors.”*

**Reviewer #1, Comment #6:** Method - I could not find the list of motivations for social distancing and social distancing behaviours in this section. The authors should here refer to Table 2 and Table 3 from the results section to allow the reader to know the motivations for social distancing and social distancing behaviours.

**Our response to Reviewer #1, Comment #6:** please see our response to Comment # 1 above.

**Reviewer #1, Comment #7:** Method - P. 9 The authors conceptualised adherence to social distancing as “always” endorsing the behaviour (coded as “1”) and nonadherence as behaviour endorsed less often than “always”, including “never”, “sometimes”, or “often” response choices (coded as “0”). It would be good to specify why “never” was treated the same way as “sometimes” and “often” and what this could imply for your results. **If the reason is purely methodological, then I wonder why not using a tobit model.** If conceptual, please specify. Also, please give an example of a behaviour where the “not applicable” option could be used.

**Our response to Reviewer #1, Comment #7:** We added the following text to the Method section, Outcome variables subsection:

- *“This dichotomy was created based on conceptual reasons, given that social distancing is effective only when practiced consistently. “*

**Reviewer #1, Comment #8:** Method - Does the model include variables for country of residence? Because countries took different approaches (even within the same category ‘moderate rules’ / ‘strict rules’, there are differences in the measures adopted), the behaviours may also vary by country.

**Our response to Reviewer #1, Comment #8:** To clarify how we used ‘country of residence’ as a predictor variable in regression analyses, we added the following text to the Data Analysis section:

- *“In regressions analyses, the variable country of residence was dichotomized into countries with strictly enforced guidelines for social distancing i.e., lockdown enforced by government authorities or police, versus countries with recommended guidelines for social distancing, but not enforced by government organizations or police. The coding sheet we used to collect information about national policies is available at* [*https://doi.org/10.17605/OSF.IO/YX67C*](https://doi.org/10.17605/OSF.IO/YX67C)*.”*

The dichotomization of variable “country of residence “was also previously included in the ***Note*** for Table 4.

**Reviewer #1, Comment #9:** Method - P. 9 The authors say: “During data collection, recommendations and policies for social distancing differed by region or country but did not change within one region or country, hence our regression models did not account for timing of survey completion.”. If, on the one hand, recommendations and policies did not change, on the other, the number of cases and deaths have increased over the period of analysis and this might have changed people’s behaviours by for example increasing their adherence to the social distancing measures. It would be good if the authors could account for the passage of time in their analysis.

**Our response to Reviewer #1, Comment #9:** In addition to the above-referenced text in the method section, we had also mentioned in the Data Analysis section that analyses comparing the report of social distancing behaviours across the three weeks of data collection, with results of this analysis reported in Table 3.

To clarify the comparison of social distancing behaviours across the three weeks of data collection, we replaced:

- *“Chi square tests were used to compare responses on the outcome variables obtained in the first, second and third week of recruitment”- with*
- *“Chi square tests were used to compare adherence to social distancing behaviors, obtained in the first, second and third week of recruitment.”*

**Reviewer #1, Comment #10:** Results - The results section is very difficult to follow because the results are presented as if they were reported on a presentation with bullet points. The whole section is organised in a similar fashion: “Endorsement rates for the four sets of motivations “for” (facilitators) and “against” (barriers) social distancing are included in Table 2. Highest endorsement rates were found for the following facilitators of social distancing: “I want to protect myself” (84%) and “I want to avoid spreading the virus to others” (83%) (individual-level facilitators); “I want to protect others” (86%) and “I feel a sense of responsibility to protect our community” (84%) (interpersonal-level); “My workplace/ school recommended we practice social distancing” (54%) and “My workplace /school conducts operations remotely” (51%) (organizational-level); “Restaurants in my area are closed for eating-in” (95%) and “Community centers and recreational facilities in my area are closed” (94%) (community-level).” The authors should find a better way to present the results because the way it stands now is not ok.

**Our response to Reviewer #1, Comment #10:** We edited the results section for clarity and readability.

- *Page 10/11: The results section presenting endorsement rates relating to facilitators and barriers to social distancing was entirely restructured to improve comprehension. For instance, we grouped motivations “for” and “against” according to the four levels of analysis (i.e., individual, interpersonal, organizational and community). We also increased the legibility of the section by eliminating the use of information in brackets and quotation marks as much as possible.*

**Reviewer #1, Comment #11:** Discussion - The organizational-level motivations against social distancing stand out for having the lowest endorsement rates, i.e. “My workplace/ school recommended we practice social distancing” (54%) and “My workplace /school conducts operations remotely” (51%). Can the authors speculate why we get such low rates in this cluster?

**Our response to Reviewer #1, Comment #11:** We added the following text (in bold) to the discussion:

- *“Notably, adherence to behaviours taxed by external circumstances (e.g., institutional policies or geographic location) including working remotely or ordering meals online, was lower,* ***which could be due to reduced motivations driven by extrinsic factors or simply a result of fewer policies promoting social distancing within institutions or certain location”***

**Reviewer #1, Comment #12:** Discussion, Limitations section: The implications of the limitations should be discussed in the paper. The authors mention three limitations of their study, but do not discuss their implications. Among them, the issue related to the sample selection is the most important. The authors cannot do much about it, and I think that they have been clear about the fact that the sample is not representative of the general population. On the other hand, I think the authors should at least discuss what are the implications of using this sample. If possible and sensible, I recommend having a table that compares the sample characteristics to the characteristics of the general population; this way we could at least know how the sample differs from the population.

**Our response to** **Reviewer #1, Comment #12:** We believe that we had adequately addressed the limitations of this study as well as the implications of stated limitations. We **bolded** the implications in the text below.

Discussion section, limitations subsection:

- *“There are limitations to this study, including the use of a convenience sample (i.e., recruited on social media) and homogeneity of sample characteristics (i.e., 84% female, >70% completed at least a bachelor’s degree),* ***which might affect the generalizability of our findings to predominantly male samples, more diverse samples, or individuals without easy access to the internet and social media platforms (Facebook, Twitter).*** *The cross-sectional design allowed for testing of associations between predictor and outcome variables at one point in time, but* ***longitudinal predictions could not be made.*** *Lastly, the outcome variables, social distancing behaviours, and some of the predictor variables, including motivations for social distancing, were created by the authors specifically for this study and* ***their psychometric properties are currently unknown****. As such, it is possible our measures did not cover an exclusive range of behaviors and/or motivations about social distancing. “*

Also, given that we recruited participants from various countries, we consider to be impossible to state what the characteristics of a “general population” sample would be. We believe that stating that we used a ‘convenience sample’ is self-explanatory and a limitation of the current study that cannot be further addressed.

**Minor points**

**Reviewer #1, Comment #14:** The authors say one of the social distancing measures is “maintaining a 2-metre distance between self and others when in public”. It would be good to specify what ‘when in public’ means. In particular, does social distancing apply to the private sphere too? For example, if I am visiting my parents at their home, do we still have to maintain the distance? Also, social distancing varies across countries from two metres down to one metre. It would be good to either be more general and say “at least a 1-metre distance”, or if the 2-metre rule is kept be more specific about where.

**Our response to Reviewer #1, Comment #14:** We updated the referenced statement to reflect varying recommendations for physical distance:

- *“…maintaining a 1 or 2-metre distance between self and others when in public, depending on the country”.*

**Reviewer #1, Comment #15:** P.5 The authors say: “Since the World Health Organization (WHO) declared COVID-19 a pandemic on March 11, 2020, national and international public health agencies proposed several measures to contain or mitigate the virus transmission ranging from complete quarantine of the population of an entire region, as in Wuhan, China (virus containment) to various degrees of social distancing measures coupled with rigorous personal hygiene (e.g., washing hands frequently and thoroughly, avoiding touching the eyes, nose, and mouth, coughing and sneezing into the elbow; wearing face masks when in public) in Canada, the United States, and Europe (mitigation of transmission).”. *The response was not the same across Europe, in fact some European governments imposed a national quarantine. On 9 March, i.e. two days before WHO announced COVID-19 outbreak a pandemic, the Italian government imposed a national quarantine like the Chinese government did in Wuhan. Italy was not the only one, others followed, e.g. Greece.*

**Our response to Reviewer #1, Comment #15:** The reviewer is correct to acknowledge that various countries responded differently to the pandemic. For the purposes of our work, we grouped the countries into those with strictly enforced guidelines for social distancing i.e., lockdown enforced by government authorities or police, versus countries with recommended guidelines for social distancing, but not enforced by government organizations or police. Please also see our response to Comment #8 above.

**Reviewer #1, Comment #16:** P.6 “Finally, given that men are more likely to die from COVID” should be “Finally, given that men are more likely to die from COVID-19”.

**Our response to Reviewer #1, Comment #16:** We made this correction.
