# Supplementary material for: Barriers and facilitators of adherence to social distancing recommendations during COVID-19 among a large international sample of adults
Source: PLoS One. 2020 Oct 7;15(10):e0239795. doi: 10.1371/journal.pone.0239795 (PMC7540845; doi:10.1371/journal.pone.0239795)
Supplement: Attachment — Submitted filename: Reviewers Comments.R2.2020.09.11.docx [file pone.0239795.s003.docx]

**Response to Reviewers’ Comments, R2: PONE-D-20-13845**

Dear Drs. Capraro and Andriano,

Thank you for reconsidering our manuscript and providing more suggestions for improvement. As with the previous revision, we are grateful for the opportunity to revise our work. Please see below our detailed responses to the Reviewer’s comments, in addition to a summary of changes made to the manuscript.

**REVIEWER #1: COMMENTS – PONE-D-20-13845 - LILIANA ANDRIANO**

“This is my second review of this manuscript. Below I comment on the authors’ responses to each point I made in my first review and include some new comments. Original comments are in black text, and comments part of this review are in **blue** text.”

**Comment #1:** “Background - The authors say “Finally, given that men are more likely to die from COVID and older adults are at higher risk of being infected by Sars-Cov-2, it is likely that gender and age could differentially impact adherence to social distancing behaviours.” Can the author cite studies showing that men are more likely to die from COVID-19 and older adults are at higher risk of being infected by Sars-Cov-2? Also, I wonder if the fact that men die more than women and that older adults are more likely to be infected compared to their younger counterparts is the only reason why gender and age could impact adherence to social distancing behaviours differently. It would be good if the authors could expand on the other plausible reasons why we could find heterogeneous results. What does the literature tell about women behaving more cautiously than men, and why may adherence to preventative health behaviours vary by sex and age? Also, if men are more likely to die from COVID-19, they are likely to be more adherent to social distancing recommendations, but even the authors find that men are less adherent to social distancing recommendations.

**Partially addressed. The authors have not commented on the last issue ‘If men are more likely to die from COVID-19, they are likely to be more adherent to social distancing recommendations, but even the authors find that men are less adherent to social distancing recommendations’, and I think they could provide some explanations for why they find that men are less adherent to social distancing recommendations.**

**Our response to Comment #1:** We have added the following to the discussion of findings:

- ***“****Results from the current study suggest that men are less adherent (~30-40 % non-adherence) to social distancing recommendations compared to women (~15-30% non-adherence). These findings are consistent with another international survey which found women were more compliant to sheltering-in-place rules (44). This finding may be explained by gender-specific differences in health information speaking behaviour, where women are more likely to actively seek out and engage with health information (46) and risk tolerance and behaviour which is generally lower in women (47)*.”

**Comment #2:** Background -The authors say “In the context of the COVID-19 pandemic, it seems reasonable to assume that individual reasons to adhere to social distancing measures (e.g., desire to protect self and others) as well as external circumstances or motivators (e.g., workplace/school conducted remotely) contribute to engagement in and adherence to preventative behaviours, such as social distancing. In addition, individual characteristics, such as demographic and psychological profile (educational level, health literacy, anxiety/stress, empathy towards others) might also play a role in adherence. Finally, given that men are more likely to die from COVID and older adults are at higher risk of being infected by Sars-Cov-2, it is likely that gender and age could differentially impact adherence to social distancing behaviours.” One factor that is lacking in this paragraph is the family. An individual is part of a family. An individual may live with their partner, their kids, etc. They may also live with the most vulnerable people in this pandemic, such as their old parents or a partner with a pre-existing illness. The family composition may be an important socio-demographic predictor of social distancing behavioural outcomes. It would be good to include this factor in the analysis, or, if not available, at least discuss it in the background section.

**Partially addressed. A discussion of this factor is still lacking in the background section. The authors should consider adding this factor to the paragraph discussing the sociodemographic variables that might play a role in adherence to social and physical distancing.**

**Our response to Comment #2:** We would like to thank the reviewer for noticing this oversight. We have added a discussion regarding both household size and composition to the background section. This paragraph now reads:

- “In the context of the COVID-19 pandemic, it seems reasonable to assume that motivations or individual reasons to adhere to recommendations about social distancing (e.g., desire to protect self and others) as well as external circumstances or motivators (e.g., workplace/school conducted remotely) contribute to engagement in and adherence to preventative behaviours, such as social distancing. *These motivations also likely interact with various* sociodemographic variables, such as gender, age, socioeconomic and minority status, health status, *and household size and composition. For instance,* it has been found that mortality from and severity of COVID-19 is higher among men (27,28), older individuals (28,29), those with predisposing conditions (29), and racial minorities. In a Southern state of the United States with a population of 31% Black, hospitalization rate was 77% and mortality rate was 71% for Blacks compared to whites (30). *Socioeconomic status also intersects with size of the household, with economically disadvantaged individuals being more likely to live in overcrowded housing, limiting the ability to socially distance (31). Further, health status of other individuals in the household (e.g., living with family members that are more vulnerable to COVID-19 infection and health consequences such as older people or individuals with pre-existing health conditions) may also have an impact on motivation and social distancing behaviour.*”

**Comment #3:** Method - P. 9 The authors conceptualised adherence to social distancing as “always” endorsing the behaviour (coded as “1”) and nonadherence as behaviour endorsed less often than “always”, including “never”, “sometimes”, or “often” response choices (coded as “0”). It would be good to specify why “never” was treated the same way as “sometimes” and “often” and what this could imply for your results. If the reason is purely methodological, then I wonder why not using a tobit model. If conceptual, please specify. Also, please give an example of a behaviour where the “not applicable” option could be used.

**Partially addressed. The authors have not commented on the last issue: “please give an example of a behaviour where the “not applicable” option could be used.”**

**Our response to Comment #3:** We added the following text to the Method section, Outcome variables subsection:

- *“Items also included a ‘Not applicable’ option to account for the possibility that not all social distancing behaviours are necessary or relevant for all (e.g., working from home for jobs that cannot be completed remotely or completing coursework remotely for non-students).”*

**Comment #4:** Method - Does the model include variables for country of residence? Because countries took different approaches (even within the same category ‘moderate rules’ / ‘strict rules’, there are differences in the measures adopted), the behaviours may also vary by country.

**Partially addressed. Measures taken were different across countries even when countries fell under the same category, and this should be taken into account in the model, are results robust to including country fixed effects (as opposed to countries with strictly enforced guidelines for social distancing etc.)?**

**Our response to Comment #4:** We appreciates the reviewer’s comment on this important matter and acknowledge this as a limitation of our survey questionnaire. WE added the following text to the limitations section:

*“In hindsight, we believe it would have been beneficial to ask respondents to identify the levels of restrictions in their area of residence, as opposed to inferring the restrictions based on their country of residence.”*

In addition, we added some needed clarification about how we used ‘country of residence’ as a predictor variable in regression analyses, we added the following text to the Data Analysis section:

- *“In regressions analyses, the variable country of residence was dichotomized into countries with strictly enforced guidelines for social distancing i.e., lockdown enforced by government authorities or police, versus countries with recommended guidelines for social distancing, but not enforced by government organizations or police. The coding sheet we used to collect information about national policies is available at* [*https://doi.org/10.17605/OSF.IO/YX67C*](https://doi.org/10.17605/OSF.IO/YX67C)*.”*

We also noted how we dichotomized the variable “country of residence “ in the ***Note*** for Table 4.

**Comment #5:** Method - P. 9 The authors say: “During data collection, recommendations and policies for social distancing differed by region or country but did not change within one region or country, hence our regression models did not account for timing of survey completion.”. If, on the one hand, recommendations and policies did not change, on the other, the number of cases and deaths have increased over the period of analysis and this might have changed people’s behaviours by for example increasing their adherence to the social distancing measures. It would be good if the authors could account for the passage of time in their analysis.

**Not addressed. The passage of time should be taken into account in the model estimation, or the authors should at least check that results are robust to this inclusion.**

**Our response to Comment #5:** Respectfully, we believe that we adequately addressed this point in the first revision. To summarize our point included in the previous revision, there were minimal to no differences found between responses for the outcome behaviours between week, week2 and week 3 of data collection- so we decided not to include a covariate variable for “time”.

**Comment #6:** Discussion, Limitations section: The implications of the limitations should be discussed in the paper. The authors mention three limitations of their study, but do not discuss their implications. Among them, the issue related to the sample selection is the most important. The authors cannot do much about it, and I think that they have been clear about the fact that the sample is not representative of the general population. On the other hand, I think the authors should at least discuss what are the implications of using this sample. If possible and sensible, I recommend having a table that compares the sample characteristics to the characteristics of the general population; this way we could at least know how the sample differs from the population.

**I agree with the authors that they had already discussed their limitations, as I also acknowledged in my previous comment. My point was about the implications of the limitations that could be further discussed. For example, what does the fact you are missing part of the population mean in terms of your results, e.g. how do you think the endorsement rates might change?**

**Our response to Comment #6:** Respectfully, we believe we addressed this point adequately in our previous revision. We feel strongly against speculating on the specific consequences of the study limitations, given that we do not have data to support such claims. With the aims of this study being primarily descriptive, exploratory, while using a convenience sample, the implication is that results need to be interpreted with these limitations in mind. We included our response to the previous revision below, for reference:

**OUR RESPONSE INCLUDED IN REVISION 1:**

*******

**Our response to** **Reviewer #1, Comment #12:** We believe that we had adequately addressed the limitations of this study as well as the implications of stated limitations. We **bolded** the implications in the text below.

Discussion section, limitations subsection:

- *“There are limitations to this study, including the use of a convenience sample (i.e., recruited on social media) and homogeneity of sample characteristics (i.e., 84% female, >70% completed at least a bachelor’s degree),* ***which might affect the generalizability of our findings to predominantly male samples, more diverse samples, or individuals without easy access to the internet and social media platforms (Facebook, Twitter).*** *The cross-sectional design allowed for testing of associations between predictor and outcome variables at one point in time, but* ***longitudinal predictions could not be made.*** *Lastly, the outcome variables, social distancing behaviours, and some of the predictor variables, including motivations for social distancing, were created by the authors specifically for this study and* ***their psychometric properties are currently unknown****. As such, it is possible our measures did not cover an exclusive range of behaviors and/or motivations about social distancing. “*

Also, given that we recruited participants from various countries, we consider to be impossible to state what the characteristics of a “general population” sample would be. We believe that stating that we used a ‘convenience sample’ is self-explanatory and a limitation of the current study that cannot be further addressed.

*******

**Comment #7:** P.5 The authors say: “Since the World Health Organization (WHO) declared COVID-19 a pandemic on March 11, 2020, national and international public health agencies proposed several measures to contain or mitigate the virus transmission ranging from complete quarantine of the population of an entire region, as in Wuhan, China (virus containment) to various degrees of social distancing measures coupled with rigorous personal hygiene (e.g., washing hands frequently and thoroughly, avoiding touching the eyes, nose, and mouth, coughing and sneezing into the elbow; wearing face masks when in public) in Canada, the United States, and Europe (mitigation of transmission).”. *The response was not the same across Europe, in fact some European governments imposed a national quarantine. On 9 March, i.e. two days before WHO announced COVID-19 outbreak a pandemic, the Italian government imposed a national quarantine like the Chinese government did in Wuhan. Italy was not the only one, others followed, e.g. Greece.*

**Not addressed. I would suggest the authors say “…in Canada, the United States, and some European countries (mitigation of transmission).”**

**Our response to Comment #7:** We acknowledge the reviewer’s point and have changed the wording of this sentence as suggested. The sentence now reads:

- ***“****In Canada, the United States, and some European countries, these range from virus containment strategies (e.g., complete quarantine of the population of an entire region, as in Wuhan, China) to mitigation of transmission through various degrees of measures designed to keep physical distance between individuals (i.e., social/physical distancing), coupled with rigorous personal hygiene (e.g., washing hands frequently and thoroughly, avoiding touching the eyes, nose, and mouth, coughing and sneezing into the elbow) and wearing face masks when in public.”*
